# Seasonality of brain function: role in psychiatric disorders

**DOI:** 10.1038/s41398-023-02365-x

**Published:** 2023-02-22

**Authors:** Rui Zhang, Nora D. Volkow

**Affiliations:** Laboratory of Neuroimaging, National Institute on Alcohol Abuse and Alcoholism, National Institutes of Health, Bethesda, MD 20892-1013 USA

**Keywords:** Neuroscience, Psychiatric disorders

## Abstract

Seasonality patterns are reported in various psychiatric disorders. The current paper summarizes findings on brain adaptations associated with seasonal changes, factors that contribute to individual differences and their implications for psychiatric disorders. Changes in circadian rhythms are likely to prominently mediate these seasonal effects since light strongly entrains the internal clock modifying brain function. Inability of circadian rhythms to accommodate to seasonal changes might increase the risk for mood and behavior problems as well as worse clinical outcomes in psychiatric disorders. Understanding the mechanisms that account for inter-individual variations in seasonality is relevant to the development of individualized prevention and treatment for psychiatric disorders. Despite promising findings, seasonal effects are still understudied and only controlled as a covariate in most brain research. Rigorous neuroimaging studies with thoughtful experimental designs, powered sample sizes and high temporal resolution alongside deep characterization of the environment are needed to better understand the seasonal adaptions of the human brain as a function of age, sex, and geographic latitude and to investigate the mechanisms underlying the alterations in seasonal adaptation in psychiatric disorders.

## Introduction

Adapting to environmental changes such as light-dark cycles are critical for survival of many species including humans [1]. Modern humans emerged close to the equator, where the day and night are equally long (12 h/12 h pattern) and constant throughout the year [2]. During early out of Africa migration, modern humans spread across continents including high-latitude areas with substantial seasonal variations in photoperiods. A geographically explicit model suggests that genetic adaptions of the human circadian clock to daylength latitudinal variations might relate to the susceptibility to mood disorders [3]. Indeed, the prevalence of psychiatric disorders including seasonal affective disorders (SAD) [4, 5], major depression [6], schizophrenia [7], and suicide attempts in bipolar disorder increases with latitude [8]. Greater seasonality in depressive symptoms is reported in high-latitude regions than in countries closer to the equator [9]. Malfunction of the biological adaptions to environmental challenges e.g., prominent light changes in high-latitude regions might increase the vulnerability to certain psychiatric disorders [3].

Many environmental variables apart from light vary across latitudes including changes in temperatures, UV radiation and allergens, and viral exposures among others. However, changes in photoperiod have been suggested to a primary contributor to these genetic adaptations [3]. Humans are very sensitive to light even with low intensities as during twilight transitions [10]. Findings from well-controlled laboratory human studies support that our internal clock adapts to changes in daylength. Specifically, after chronic exposure to artificially induced day-night cycles in laboratory settings, the endogenous circadian rhythms adjusted to the experimental conditions [11]. An early study performed between 1964–1979, reported preserved seasonal patterns of circadian rhythms in men kept isolated from external cues conditions. This suggested that circadian rhythms are entrained to seasonal changes in daylength and that there is imprinting of biological clocks to the light-dark cycle to which they had been previously exposed [12].

Patients suffering from psychiatric disorders display dysfunctions in behavior, emotion and cognition that significantly impair their social, occupational or interpersonal functioning [13]. Seasonal patterns of mood and behavior are usually assessed with questionnaires that screen for neuropsychological (mood, energy, social activity, sleep) and metabolic (appetite, weight) factors [14]. In psychiatric disorders, seasonality is observed (see systematic review Geoffroy et al., 2014 for bipolar disorder and Goimbra et al., 2016 for suicide attempts) [15, 16] and a stronger global seasonality score was associated with more severe phenotypes [14]. While season-related social factors and stressors e.g., school schedules, holidays can affect symptoms, compelling evidence suggests that biological processes play a critical role in the observed seasonality.

Seasons influence various biological pathways including gene transcription, neurotransmitters and neuropeptides and immune [17], metabolic and neuroendocrine processes [18, 19]. However, we still don’t know how biological adaptations affects seasonal patterns in mood and behavior, whether stronger biological response to seasonal changes has beneficial effects on mood stabilization and why some people experience greater seasonality than others with negative consequences to their daily life and functioning. Therefore, we review existing studies to identify potential mechanisms that could explain seasonality in psychiatric symptoms as well as directions for future research. Here we mainly focus on brain adaption since across tissues brain is among those that exhibited the highest seasonality in transcriptomes [20]. We structured this review by first describing seasonal patterns in psychiatric disorders; next, we summarize evidence of seasonal fluctuations in neurotransmitters, in brain function and structure; then based on the strong evidence for seasonal variations in immune function [17, 20], we discuss how the immune system can affect the brain and influence emotions. Given the large impact of light on circadian rhythms, we then summarized the evidence of the role of circadian rhythms in seasonal modulation and reviewed the factors that contribute to individual differences in seasonal variations. Finally, we discussed current research gaps and future directions.

## Seasonality in psychiatric disorders

**Fig. 1:**
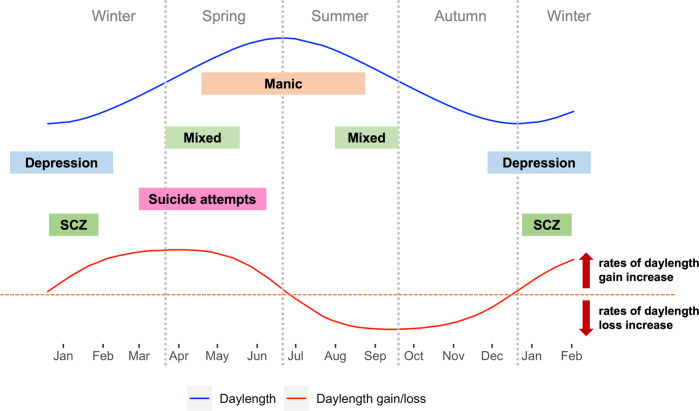
Peaks of psychiatric symptoms in relationships to daylength and day-to-day daylength gain/loss. Blue curve: daylength changes throughout the year in the northern hemisphere. Red curve: day-to-day daylength gain/loss throughout the year in the northern hemisphere. Red dashed line: threshold indicating there is no change in daylength compared to the previous day. Curve above the dashed red line indicates days that have longer daylength than the previous day, while curve below the dashed red line indicates days that have shorter daylength than the previous day. The peak around late March is the day with greatest daylength gain, the nadir around late September is the day with greatest daylength loss. Mixed: mixed symptoms in bipolar disorders; SCZ: Schizophrenia hospitalizations & first-episode onset.

Daylength and the rate of daylength changes have been proposed to underlie the seasonal fluctuations of some psychiatric symptoms. In the Northern Hemisphere for example, days are longest at the summer solstice in June and shortest at the winter solstice in December, while rates of daylength increases peak at the March/Spring equinox and rates of daylength decreases peak at the September/Autumn equinox (Fig. 1).

For affective disorders (major depression, Bipolar I & II), (hypo)manic episodes usually peak in spring/summer with a minor peak in autumn, while depressive episodes peak in winter and mixed episodes peak in early spring or mid/late summer [15, 21, 22]. It is estimated that around 10–22% of patients show seasonal emergence/exacerbation of symptoms and are classified as SAD. However the prevalence is probably underestimated since very often seasonality is not assessed [23]. Notably, greater seasonality of symptoms was associated with more severe depression and mania and higher number of relapses [14, 24]. Patients with major depression or bipolar I disorder who showed greater seasonal patterns reported higher levels of suicidal ideation and attempts [25, 26]. Atypical depressive and somatic symptoms such as hypersomnia, hyperphagia, psychomotor retardation, fatigue and reduced physical activity are more frequent in patients with SAD than without [27]. For schizophrenia, seasonality is less extensively studied compared to affective disorders. Several studies from both the southern [28, 29] and northern hemispheres [30, 31] consistently show an association of the time of admission or onset of first-episode of schizophrenia with the short photoperiod (peak in winter), while one study reported an additional peak in June. However it is unclear whether positive symptoms such as hallucinations, delusions or negative symptoms such as flattened affect, loss of motivation and social withdrawal, drive these admissions [32].

Psychiatric disorders with a seasonal pattern are considered at greater risk for suicidal behaviors [15, 33, 34]. Suicides and suicide attempts peak in the spring/summer months [16, 35] and are more prominent among people with mood disorders than without [35] and increase with distance to the equator suggesting a critical role of daylight changes [16]. In the US, intentional drug overdose deaths have a positive linear relationship with daylength [36, 37]. Furthermore, not only long daylength but also rapid changes in daylength might increase suicide rates, which could explain the repeatedly observed Spring peaks. A cross-national study documented that pronounced changes in solar insolation from winter to summer months appear to be a significant risk factor for suicide attempts in bipolar I disorder [8]. Interestingly, Swedish studies showed a Spring peak of suicides in patients with alcohol use disorder [38] whereas an Autumn peak in patients with severe depression [39]. To better serve the suicide prevention, it would be important for future studies to investigate whether seasonality in suicide attempts differ across psychiatric disorders. Furthermore, despite the overall pattern with a peak in spring/summer, countries vary in the degree of seasonality indicative of the contribution of social and cultural influences [40].

Emerging studies leverage large datasets of the internet to investigate seasonality in mental problems in the population. Key words that searched for various mental illness on Google over a period of 5 years, showed peaks in winter in both northern and southern hemispheres [41]. Across the globe, seasonal rhythms in mood are also seen from social media posts and associated with changes in daylength. Positive affect is higher when days becomes longer and is highest when the changes in daylength are greatest, while seasonal changes in negative affect were not found [42]. Consistent with this, in a representative population sample from Switzerland, among people who did not meet seasonal criteria, seasonal rhythms were present with better well-beings/psychological symptoms (mood, social contact, energy) and less vegetative symptoms (sleep, appetite, weight) in spring/summer than autumn/winter but to a lesser extent than among those who met criteria [43].

In sum, though most studies are retrospective and cross-sectional. The samples sizes from surveys of populations are larger than from clinical studies. Most survey studies are based on the calendar for seasonal classification but future use of the astronomical calendar, which considers daylength variations and involves more time measurement might increase the sensitivity for studying the relationship between day-night cycles and psychiatric symptoms. In general, seasonal patterns in psychiatric disorders are consistently observed across countries, which points to underlying mechanisms beyond those driven by social and cultural components. The next sections summarize findings on brain changes throughout the seasons and how they relate to psychiatric symptoms.

## Seasonal changes in neurotransmitters

Seasonal variations have been reported for multiple neurotransmitter systems. Serotoninergic (5-HT) [44, 45] and dopaminergic (DA) systems [46–48] are the two most studied because of their essential role in mood, cognition and reward [49–53].

### Dopamine

Postmortem studies have examined midbrain DA neurons in people who died in winter vs summer and found that tyrosine hydroxylase (TH, rate-limiting enzyme in DA synthesis) and DA transporter (DAT, which are involved in DA reuptake into the presynaptic terminal limiting DA signaling) immunoreactivity in neurons were qualitatively lower in winter than in summer [46]. Consistently, a positron emission tomography (PET) study showed lower striatal DAT availability measured with [123I]beta-CIT in symptomatic depressed patients with SAD than in healthy controls [54]. TH and DAT dynamically regulate the homeostasis of the DA system. Low DA synthesis from reduced TH expression could be compensated by downregulation of DAT to increase duration of DA in the extracellular space and vice versa. A postmortem study reported higher levels of DA or DA metabolites in fall/winter compared to spring/summer in hypothalamic tissue of healthy controls and in ventral striatal tissue of schizophrenia patients [55]. In line with this, CSF findings from healthy adults, schizophrenia patients and Alzheimer’s patients documented an increase of DA metabolite concentrations in fall/winter compared to spring/summer [56, 57]. PET studies have documented higher striatal presynaptic DA levels measured with [18 F]-DOPA [47, 58] and lower striatal D2/D3 receptor availability measured with [123I]-IBZM in winter [48], which could either reflect increased DA levels that compete for binding with [123I]-IBZM or reduced levels of D2/D3 receptors. In winter with the shorter daylengths, melatonin release is prolonged [59], which could help explain this seemingly conflicting findings. Specifically, preclinical studies have reported that while melatonin inhibits postsynaptic striatal DA signaling [60], it promotes presynaptic DA neuronal integrity [60, 61]. In contrast to PET findings, studies using spontaneous eye blink rate, as an indirect measure for DA signaling, showed higher eyeblink rates in the spring/summer than in the fall/winter both for healthy participants [62] and patients with schizophrenia [55]. However, the evidence of baseline eyeblink rates as a biomarker of brain DA activity is not consistent [63, 64].

### Serotonin

In postmortem human brain, 5-HT levels in the hypothalamus were lowest in the winter [65]. Consistently, a study that measured blood samples from 101 healthy men reported the lowest turnover of 5-HT in winter, which increased with extended bright light exposure [44]. Using PET, higher 5-HT_1A_ receptor availability measured with [11C]-WAY-100635 in 5-HT projection regions in frontal, temporal, insular and cingulate cortices and in amygdala and hippocampus, where 5-HT_1A_ receptors are mostly postsynaptic [66], were associated with longer daylengths [45] and total light intensity [67]. In contrast, higher serotonin transporter (SERT, responsible for 5-HT reuptake into presynaptic neurons) availability measured with [11C]-DASB in prefrontal cortex, striatum, thalamus and midbrain were associated with shorter daylengths and peaked in the fall/winter in healthy participants [68]. However, this observation was not confirmed in a within-subject design SPECT study that used [123I]ADAM [69]. Variations in 5-HT_1A_ signaling and SERT could relate to seasonal mood changes as antidepressants are believed to exert their therapeutic effects in part by blocking SERT and increasing postsynaptic 5-HT_1A_ signaling [70, 71]. For patients with SAD, SERT availability in the brain (including prefrontal and anterior cingulate cortices) were upregulated in winter to a greater extent than in healthy controls [72, 73] and it was proposed that the development of depressive symptoms in winter in SAD patients might reflect failure to downregulate SERT [72]. Individuals who are resilient to SAD downregulate SERT in winter, which was thought to be beneficial for maintaining stable synaptic 5-HT level [74]. Cortical regions in SAD resilient individuals that exhibited seasonal adjustments of SERT levels included the right posterior medial, left inferior temporal and occipital cortices and angular gyrus [74]. A recent PET study examined monoamine oxidase A (MAO-A), an enzyme which degrades amine neurotransmitters including DA, 5-HT in healthy controls and patients with SAD with repeated measures in fall/winter and spring/summer. Although patients with SAD did not differ from healthy controls in cerebral MAO-A, they show reduced seasonal dynamics in MAO-A [75]. In healthy controls, MAO-A decreased from fall/winter to spring/summer, which was not seen in SAD patients [75]. Interestingly, 3-week bright light therapy significantly reduced MAO-A levels in the brain of SAD patients suggesting an important role of light in regulating MAO-A [75].

In sum, there is strong support for seasonal variations in 5-HT and subcortical DA signaling in the brain of healthy controls and of individuals with SAD. Yet, the findings are hard to interpret considering that different measures (direct vs indirect), targets (metabolites, synthesis, receptor, transporter) and regions (CSF, cortical, subcortical) have been assessed by the various studies. Further, 5-HT and DA are not independent systems, and they have strong interactions with each other. For example, animal studies show 5HT_1A_ receptor activation stimulates DA release in the prefrontal cortex while it inhibits DA release in the striatum [76]. According to human studies, cortical SERT and striatal DAT seemingly display opposite seasonal patterns that are both associated with SAD depression symptoms [46, 54, 68, 73]. Thus, the ratio and balance between DA and 5-HT are likely to be relevant for the presentation and severity of psychiatric symptoms. Additionally, there is evidence for blunted seasonal regulation of neurotransmitter system e.g., SERT and MAO-A [72, 75] in SAD patients. Dysregulation of 5-HT and DA systems has been assumed to underlie various psychiatric disorders [77, 78]. However, seasonal variations of 5-HT and DA still need to be examined in psychiatric disorders other than mood disorders. Beyond 5-HT and DA, accumulating evidence has supported seasonal fluctuation in other neurotransmitter systems. A recent study reported a U-shaped relationship between daylength and mu opioid receptor availability in humans [79]. Animal studies have further revealed positive and negative correlations of daylength with norepinephrine [80] and acetylcholine [81], respectively, which have not been examined in humans.

## Seasonal changes in brain function and structure

In contrast to extensive studies on seasonality at the biochemical level, very few studies have investigated seasonal effects on brain activity, which is strongly modulated by neurotransmitters. A cross-sectional study from Belgium demonstrated seasonal variations of cognitive brain responses in twenty-eight young healthy participants after living without any seasonal cues for 4.5 days suggesting that there might be a”photic memory” for the photoperiod to which the participants were exposed prior to the study [82]. The authors reported different seasonal patterns for various cognitive components: While basic attentional processes were associated with daylength, higher level executive brain responses covaried with day-to-day daylength variations [82]. In young adults in the US, the amplitude of P300 event-related brain potential, which reflects processes involved in high-level cognition such as evaluation and decision-making, was larger in subjects tested during spring/summer than in fall/winter [83, 84]. Although patients with psychiatric disorders show reduced performance in various cognitive domains compared to healthy controls [85, 86], whether their cognitive deficits vary across seasons remains unclear. Moreover, neuroimaging studies on brain activations associated with seasonal fluctuations in affective control and reward function are still lacking.

Another promising research area is that of seasonal variations in resting state fMRI, which is less affected by study-specific factors and allows comparisons across studies. Particularly, resting-state functional connectivity (RSFC) is highly correlated with brain activation patterns during task performance [87]. In a recent German study with fourteen healthy male volunteers, resting-state fMRI signal variance drops endogenously (i.e., not evoked by external cues) at times coinciding with dawn and dusk in sensory regions including the bilateral visual, somatosensory and right auditory cortices [88]. The sensorimotor network (SMN) exhibits strong recurrent connections consistent with localized processing of external stimuli [89]. Therefore, SMN could be a core cortical network that receives information from the internal clock and conveys daylength information to the rest of the brain. There have already been some observations for associations of brain network dynamics with different affective states. In bipolar disorder the shift of depressive and manic phases has been suggested to relate to the balance between default mode network (DMN) and SMN. The intrinsic brain activity was shifted toward the DMN during the depression phase that is characterized with internal thoughts and ruminations [90] and toward the SMN during the manic phase that is characterized by excessive focus on external environmental stimuli and psychomotor overexcitement [91, 92]. Longitudinal evidence further support the involvement of interoceptive-sensorimotor during the hypomania phase and of the DMN during the depression phase in bipolar disorder [93]. However, in these studies, seasonal effects were not considered, and seasonal patterns were not assessed in patients with bipolar disorders. It still needs to be confirmed whether patients with seasonal patterns showed comparable network dynamics as that in non-seasonal patients.

Bain structure studies that have studied seasonal effects focused on subcortical regions relevant for emotional regulation using large datasets. Cross-sectional studies done in healthy adults from the UK and the US documented positive associations of daylength with volumes in subcortical regions including hippocampus [94, 95], amygdala [96] and brainstem [97], which are regions that display seasonal variations in 5-HT signaling [45]. According to evidence from preclinical studies [98], cortical regions might also display volumetric seasonal changes, which need further investigation in prospective clinical studies with repeated measures. So far, to our knowledge no studies have examined seasonal effects on structural or functional connectivity in the human brain.

Together, there are multiple research gaps including neuroimaging studies on seasonal variations in brain function and structure in patients with psychiatric disorders. For this purpose, longitudinal designs with sufficient sample size and high temporal resolution to examine daylength and rates of daylength changes and compare patients and healthy controls are needed.

## Contribution of immune system to brain adaptation

Genes in the brain and gonads showed the strongest seasonal expression profiles among 46 tissues based on transcriptomic analyses in postmortem tissues of 932 donors and immunity related genes were enriched among genes that showed seasonal expression profiles, consistent with previous findings [20, 99]. The immune system has a profound pro-inflammatory transcriptomic profile during European and Oceania winter, with increased levels of soluble IL-6 receptor and C-reactive protein [17]. Interestingly, emerging evidence suggests a link between immune dysfunction and changes in brain structure and function in psychiatric disorders. Associations are reported for frontal and temporal regions that are engaged in cognitive and affective control [100–102]. Behaviorally, correlations between inflammatory biomarkers and poor cognitive performance have been observed [103, 104]. Neuroinflammation could be one potential mechanism that contributes to seasonality in psychiatric disorders. However, as of now no studies have examined seasonal changes of immune function in patients and how they differ from healthy participants. Given the immune-brain relationship seen in psychiatric disorders future studies should assess their involvement in the seasonal effects reported for frontotemporal regions and their association with cognitive and emotional symptoms. Moreover, investigation of specific immune processes that might be involved with seasonal expression of psychiatric diseases could lead to potential therapeutic interventions.

## The role of the circadian rhythm in seasonal control

Humans have intrinsic circadian rhythms that are slightly longer than 24 h (approximately 24.2 h) and exquisitely sensitive to light [105–107]. The near 24-hour oscillations can be found in almost every biological and physiological processes in human brain and body. Light is the most prominent environmental cue that entrains the endogenous circadian rhythm to the 24-h day. The suprachiasmatic nucleus (SCN), the master circadian pacemaker in the brain, receives light input and conveys timing information by regulating neuronal activity, body temperature and hormonal signals [108]. Human brain postmortem studies suggest that the SCN not only plays a role in temporal organization of near-24-hour circadian processes but also in seasonal control. The volume and number of vasopressin neurons in the SCN, which transmit photic information to the brain vary throughout the day with two peaks around twilight in young subjects [109]. This same group from the Netherlands also reported seasonal changes in subjects 6–91 years of age. The volume and number of vasopressin neurons are highest during October when the daylength becomes shorter and rates of decreases in daylength are greater, while the lowest is around June when the daylength is the longest and variations in daylength minimal [109, 110]. In addition to the peak in October, there is another smaller peak round March when the increases in daylength accelerate [110]. The annual pattern of two peaks around Spring and Autumn equinox were even more prominent when including only young subjects [109]. Together, increases in neuronal volume and number in SCN might help us optimally respond to dramatic photic transition during twilight and equinox, which is critical for the regulation of daily and annual activities.

Melatonin and core body temperature have been used to measure endogenous circadian rhythms in humans. Astonishingly, the seasonal variations of the period of core temperature mirrored the pattern of SCN morphology. The period was shorter around the spring and autumn equinox (shortest in Spring) than summer and winter [12]. In terms of rhythm timing, oral temperature peak time was earlier in December than in March or June [111]. A direct comparison of two core body temperature studies is difficult since the latter one has lower temporal resolution and oral temperature is not always accurate for assessing core body temperature [112]. Surprisingly, few studies have examined melatonin fluctuation across seasons. A French study with four measures in January, March, June and October reported higher plasma melatonin levels in June than January in young men. In contrast, in an experimental setting, the duration of melatonin secretion was shorter after exposure to the ‘Summer’ photoperiod along with shorter sleep duration in young men [59]. In extreme environment such as in an arctic latitude, seasonal changes in timing rather than duration of melatonin release was observed [113]. A delay of circadian phase was reported in winter accompanied by later sleep timing and poor sleep quality [113–115]. However, these studies are limited by their very small sample sizes (5–7 subjects in each study) and need to be replicated [116]. Together, various circadian processes such as core body temperature and melatonin release might display different seasonal profiles. The questions remain of whether distinct patterns of circadian processes contribute to the emergence of psychiatric symptoms at different times of the year, whether there is a misalignment between the various biological seasonal processes and seasonality in symptoms of mood and other behavior. Specifically, whether SCN responses to equinox in Spring and Autumn affect the psychiatric symptoms emerging in Summer and Winter. To answer these questions, more rigorous studies are required. Both period and timing of rhythms could be relevant for understanding how circadian processes participate in seasonal adaptions and expanding from two-time point measures i.e., winter vs summer to higher temporal measures are required to capture the complex seasonal dynamics.

The seasonal entrainment of circadian rhythms can influence brain function by modulating neurotransmission. Preclinical studies documented reciprocal connections between the SCN, the dorsal raphe nucleus (main hub for 5-HT) and the ventral tegmental area and nucleus accumbens (main hubs for DA) [117–122]. Circadian patterns are seen in both DA and 5-HT activities in animals. While DA activity is higher during the active phase [123], the mRNA levels of tryptophan hydroxylase, the 5-HT rate-limiting biosynthesis enzyme, peak around the light transition [124]. The dramatic and persistent impact of photoperiod on 5-HT neurons depends on melatonin signaling [80]. Thus, the SCN can entrain 5-HT and DA signaling to the photoperiod thereby adjusting their modulatory functions to environmental changes. 5-HT and DA afferents also convey information to the SCN and modulate its activity. Both 5-HT and DA can modulate light-induced circadian phase-shifting in rodents. While 5-HT increases or decrease light-induced circadian phase-shifting depending on activated receptor subtype and location (e.g., presynaptic vs. postsynaptic), DA agonists reduce the phase-shifting effect induced by light [125]. Therefore, disrupted and imbalanced neurotransmitter systems in patients with psychiatric disorders could affect their circadian adaptations to seasonal changes [125]. Interestingly, immune factors modulate phasing of circadian clocks and therefore could contribute to circadian adaptations to seasonal changes [126]. It is possible that people with immune dysfunctions such as that reported in some psychiatric diseases might have difficulties in adjusting circadian rhythms to light-dark cycles as they vary across the seasons. Lastly, unadjusted circadian rhythms could disrupt rest-activity rhythms and reduce light exposures, which would further destabilize circadian rhythms.

## Are there adaptive benefits of seasonal entrainment?

Although there are many unknowns, current findings support the belief that greater seasonal adjustment of neurotransmitter is likely beneficial for maintaining stable mood throughout the year. This is consistent with the greater seasonal dynamics of SERT [72] and cerebral MAO-A [75] observed in healthy controls than in patients with SAD. Additionally, there is indirect evidence from studies with exposure to artificial light, which have shown to suppress seasonality of biological rhythms [127] and of sleep-wake cycles [128–130] and might increase the risks for SAD. In non-industrial societies individuals were exposed only to natural sunlight with maximal exposure in the morning and their sleep onset varied across the seasons on average 3.3 h after sunset [131]. In contrast, for urban dwellers who are exposed to bright daylight on average 3.5 h per day [132] light melatonin onset (DLMO) and sleep timing are not associated with sunrise or sunset or differ between winter and summer [128–130]. In this respect it is interesting that the Old Order Amish in Pennsylvania who live a rural life without electric light have much lower SAD prevalence than their nearby population in Maryland [133] suggesting that biological adjustment to natural day-night cycles might benefit well-beings [134]. It is likely that through millions of years of evolution, biological processes evolved to adjust to seasonal changes. Artificial light, which was first introduced in the early 1700s interferes with seasonal entrainment of biological processes that could lead to dysfunctions in mood and behavior. Still, more research is needed to examine whether failed seasonal entrainment underlies greater seasonality in patients with psychiatric disorders.

## Individual variations

**Fig. 2:**
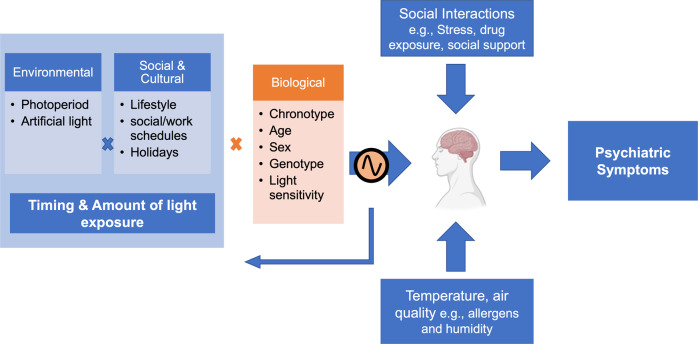
Factors that contribute to seasonal variations in mood and behavior. Exposure to light throughout the year depends on external factors such as local environment (latitude, urban vs rural) which determine how much light is available; and social and cultural factors (e.g., lifestyle, office work vs outdoor work), which impact our actual daily light exposure patterns. Further, biological factors could contribute to different brain responses to light among individuals. The seasonal effects on our brain are likely to be mediated by circadian changes. Altered circadian rhythms could in turns affect our behavior and sleep-wake cycle further influencing the timing and amount of light exposures. While brain changes across seasons can lead to altered emotional and cognitive outcomes, season-related social interactions (e.g., summer holidays, Christmas seasons) could modulate the likelihood of exposure to both disruptive (e.g., stress, alcohol and other drugs) and protective factors (e.g., social support) as well as change in our light exposure patterns. Other environmental factors that change across seasons such as temperature and air quality could also contribute to seasonal effect on our brain. The interplay between internal and external factors contribute to seasonal patterns in mood and behavior. Blue: external factors influenced by seasons; Orange: biological factors.

While studying seasonal effects at the population level is a first step, a closer examination and understanding of inter-individual differences is crucial for developing individualized interventions in psychiatric disorders. Further, there are shared risk factors for greater seasonality in mood and behavior and psychiatric disorders. We discuss external and internal factors that affect the vulnerability to seasonal changes (Fig. 2).

### Light exposure

Annual sunlight exposure patterns of individuals are affected by their local environments. Regarding geographic location, changes in daylength between winter and summer are much larger near the poles than the equator. Meanwhile, large changes of light-dark cycles induce greater challenges for internal circadian rhythms and influence mental and physical health. Greater seasonality in mood and behavior [9] and higher prevalence of SAD [4, 5] is reported in countries at high latitudes. Earlier onset of bipolar disorder is associated with greater maximum monthly increase in solar insolation at the location of onset [135]. However, this association might be dampened if participants were born in places with large amount of daylight [136]. It has been suggested that early light exposure could be beneficial for developing an internal clock that has flexibility to adapt to external circadian challenges [136] that could partially help explain some reports on the impact of season of birth on psychiatric disorders. Thus, light does not only serve as the major seasonal challenge but also has an impact on our ability to adapt to light changes [137]. Further, with globalization, more and more people start living far away from where they were born. As the internal clock is trained by early life light exposure, moving to a new environment especially with greater seasonal changes require greater biological systems adaptations which might increase the likelihood of failed seasonal entrainment [138].

### Chronotype

Greater eveningness is associated with higher self-reported seasonality score [139–141], which is independent of electrical light use or latitude [139–141]. For individuals with later chronotype, phase-delaying stimuli/evening light is expected to play a more important role than morning light. Thus, longer daylength would further delay the circadian rhythms among these individuals, whereas shorter daylength would advance the rhythm phase. Indeed, a delay of circadian rhythm was observed in spring compared to winter in adolescents, who are undergoing a developmental stage characterized by prominent phase delay [142]. This study also showed that adolescents were exposed to more light during the evening hours in spring than in winter, while daytime light exposure especially morning light exposure which is critical for phase advancing, was comparable between seasons [142]. There is evidence that greater eveningness is associated with poor mental health [143] and greater risk for depression [144]. One well-accepted hypothesis is that a stronger mismatch between endogenous circadian phase and imposed school/work schedules among later chronotypes underlies its detrimental consequences. If the mismatch is the major cause, worse outcomes such as higher level of depression would be expected in Spring/Summer than Autumn/Winter as further phase delay is expected with longer daylength in later chronotype, which still needs to be tested. Also, a better understanding of chronotype in the vulnerability to mood disorders throughout the lifespan would help target healthier policies regarding school time initiation during adolescence and to help design personalized strategies to phase advance eveningness chronotypes for those at risk from mood disorders.

### Age and sex

Self-reported seasonality is higher in younger than older adults and in women than men [139, 140]. Women have 1.5 times higher risk of seasonally related mood swings [145] and show greater seasonal variations in basic cognitive processes compared to men [84]. During winter in the South Pole, women showed more self-reported emotional problems than men [113, 146]. For psychiatric disorders, SAD is more common in youth and in women [147, 148]. The age of onset of bipolar disorder peaks between ages 15 and 24 years, with a higher prevalence of bipolar II disorder in women than in men [149]. Among patients with bipolar disorder, women appear to exhibit greater vulnerability to seasonal variations compared to men [15]. Some other findings suggest that women and men with bipolar disorder might have different seasonal patterns: While seasonal patterns for manic episodes were found for both women and men and peaked in spring/summer, a seasonal pattern for depressive and mixed episodes was only observed in women. Further, there seems to be a sex by age interaction such that young age (15–35 years) increases the likelihood for a seasonal pattern in manic and mixed episodes in women but not in men [150]. Similarly, for psychotic depression, a significant seasonal pattern was only present in women and less pronounced in older compared to younger female patients [151]. The season of birth effect that associated births in winter/spring with increased risk for schizophrenia was predominantly seen in women [152–154]. Also, the seasonal variations of binge drinking with a peak in spring/summer months [155, 156] and of intentional opioid overdoses with a peak in spring were greater in women than men [37]. Together, women with younger age show the greatest seasonal fluctuations and vulnerability to season-related psychiatric symptoms. The greater vulnerability to seasonality in women might be due to their greater sensitivity to circadian modulation than men [157]. Sex differences in seasonal effects have also been reported in neuroimaging studies. Compared to men, healthy women displayed greater seasonal fluctuations in SERT [74] and in hippocampal volumes, a relevant 5-HT projection region [94]. However, it has not been investigated whether patients with psychiatric disorders show similar sex differences. The effect of age might be interrelated with chronotype as young adults have a delayed circadian phase compared to older adults [158].

### Light sensitivity

Compared to healthy controls, hypersensitivity of circadian rhythms to light is observed in patients with SAD and bipolar disorder as well as individuals at risk for developing bipolar disorder [159]. In contrast, hypersensitivity was not found in patients with major depression [160] or in euthymic bipolar patients [161, 162]. For SAD, light sensitivity is also reported to be season-dependent such that hypersensitivity was seen in winter and hyposensitivity in summer [163]. Elevated circadian sensitivity to light might relate to delayed phase reported in both bipolar disorders and SAD [164, 165]. Further, chronotherapeutic treatments such as wearing blue-blocking glasses in the evening, bright light therapy exposure and melatonin treatment are promising interventions for treating manic [166, 167] and SAD patients [168]. In healthy adults, there are large inter-individual differences in light sensitivity of circadian rhythm such that there was an over 50-fold difference between the least and the most sensitive individuals [169]. In non-clinical populations, hypersensitivity to light was associated with mood traits related to bipolar disorder (subthreshold symptoms) particularly with hypomania but not depression [170]. Light sensitivity is also likely to partially account for the effect of age on seasonality discussed above. Adolescents, a critical age for developing various psychiatric disorders, have higher light sensitivity to short-wavelengths than adults, which might contribute to their delayed rhythm phase [171, 172].

### Genotype

There are overlapping genetic risk factors for self-reported seasonality and bipolar disorder, schizophrenia, but not for major depression [173]. 5-HT and circadian genes are the most extensively studied for explaining inherited components of seasonality [174, 175]. The short allele of the SERT linked polymorphism 5-HTTLPR was associated with greater seasonality in mood, behavior, and increased risk for SAD [176, 177]. 5-HT levels could affect circadian sensitivity to light. Administration of an acute dose of the selective serotonin reuptake inhibitor citalopram induced a 47% increase in light induced melatonin suppression [178]. Apart from 5-HT genes, core clock genes including CLOCK, ARNTL, NPAS2 and PER2 gene polymorphisms are also implicated in seasonal variations in mood, behavior and risks for developing SAD [179, 180]. Polymorphisms in the circadian clock gene *PER3*, which were associated with diurnal preferences [181] were recently linked with seasonal mood traits in transgenic mice [182]. The associations between DA genes and seasonality have been less investigated. In mice, longer photoperiod increases retinal photosensitivity, which is regulated by ocular DA signaling [183]. Genetic differences in DA system are thus likely to cause inter-individual differences in seasonality in part by modulating light sensitivity.

Moreover, melanopsin gene variation were associated with SAD [184] and changes in the timing of rest-activity rhythms in healthy individuals [185]. Melanopsin is a photopigment expressed in the retina and involved in non-image-forming responses to environmental light and thereby affecting circadian entrainment. SAD patients had a higher frequency of the homozygous minor genotype (T/T) for the missense variant rs2675703 (P10L) than healthy controls [184]. In individuals without mood disorder, sleep onset among those with the P10L TT genotype was later on longer days and earlier in shorter days and greater morningness was associated with shorter daylength [185]. Although the findings need to be interpreted with caution given the small number of individuals with the TT genotype, participants with the TT genotype show similar sleep-wake pattern as expected in later choronotypes [142].

### Season-related social interactions

Holidays e.g., summer holidays, Christmas season, typically lead to changes in social interactions. These season-related changes in social interactions could not only influence light exposure patterns but also increase the likelihood of exposure to both disruptive (e.g., drugs, stress) and protective factors (e.g., social support), thereby modulating mood and behavior. Since season-related social interactions might vary across countries and cultures, they should be considered when conducting multi-sites studies on seasonal effects.

## Conclusions and future agenda

We reviewed seasonal effects on the human brain by first summarizing neuroimaging findings on relevant neurotransmitters, intrinsic brain networks, brain structure and task-induced brain activation. Most of the evidence from published studies point to the important role of DA and 5HT systems in seasonal fluctuations of psychiatric symptoms. Beyond 5-HT and DA, the seasonal patterns of other neurotransmitter systems and neuropeptides remain to be investigated in humans. Studies on seasonal variations in brain function and structure are very sparse and most are limited by small sample sizes, cross-sectional study designs or examination of a few regions of interest. Nevertheless, studies identified brain regions and networks including SMN, subcortical 5-HT projection regions and regions involved in sustained attention that are sensitive to daylength in healthy controls. Since the identified regions are thought to be involved in cognitions and emotions, it is expected that their seasonal fluctuations would differ between psychiatric patients and healthy subjects and explain the greater seasonality observed in patients. Yet, this hypothesis still needs to be tested. While most studies on seasonal effects have examined daylength or compared fall/winter vs spring/summer, the effect of day-to-day daylength variations has been rarely investigated. A recent study highlighted the association of day-to-day daylength variations with brain activation during complex cognitive processes [82]. Studying associations with daylength variation could provide a better insight into symptoms or behaviors that peak in spring or autumn and advance our understanding of seasonal effects. Therefore, future studies with rigorous designs including high temporal resolutions and multiple measurements throughout the year are needed.

Our circadian rhythms are highly sensitive to light and are likely involved in seasonal entrainment. Longitudinal studies combining light exposure, circadian rhythms and multimodal brain measures with large sample size are required to answer several timely questions: (1) how seasonal changes in brain function and structure relate to individual’s light exposure (duration, intensity, timing) and day-to-day variations in light exposure; (2) whether brain regions with seasonal variations are associated with circadian changes, i.e., it would be relevant to identify regions that are modulated by circadian rhythms vs circadian-independent; (3) whether subjects with greater circadian variations show greater changes in brain functions and whether stronger seasonal adjustment of the internal clock (and subsequently brain function) would result in more consistent mood and behavior throughout the year; (4) whether and how patients with psychiatric disorders differ from healthy controls in light exposure patterns, and in circadian and brain adaption to seasonal changes. Finally, although light is thought be a major contributor to the seasonal effect, examining effects of other environmental factors such as temperature, air quality including allergens and humidity will help advance our understanding of this important topic.
